# High-performance inertial impaction filters for particulate matter removal

**DOI:** 10.1038/s41598-018-23257-x

**Published:** 2018-03-19

**Authors:** Xiaowei Zhang, Wei Zhang, Mingqiang Yi, Yingjie Wang, Pengjun Wang, Jun Xu, Fenglei Niu, Feng Lin

**Affiliations:** 1 Department of Electrical Engineering and Computer Science, Ningbo University, Ningbo, 315211 China; 2 National Laboratory of Solid State Microstructures, Collaborative Innovation Centre of Advanced Microstructures, and Department of Electronic Science and Engineering, Nanjing University, Nanjing, 210093 China; 3 Beijing Key Laboratory of Passive Nuclear Power Safety and Technology, North China Electric Power University, Beijing, 102206 China; 4 Microfluidic Foundry LLC, San Pablo, CA 94806 United States; 5 Department of Chemistry, Virginia Tech, VA 24061 United States

## Abstract

Airborne particulate matter (PM) is causing more and more serious air pollution and threatening the public health. However, existing air filter technologies with the easy-to-block manner can rarely meet the requirements of high-performance PM filters. Here we propose a conceptually new type of inertial impaction filters for rapidly high-efficiency PM removal. Under the airflow velocity of 8.0 m/s, the real inertial impaction filters show high PM removal efficiencies of up to 97.77 ± 1.53% and 99.47 ± 0.45% for PM_2.5_ and PM_10_, respectively. Compared with the traditional air filters reported previously, the inertia impaction filters exhibit extremely low pressure drop of 5–10 Pa and high quality factor (*QF*) values of 0.380 Pa^−1^ and 0.524 Pa^−1^ for PM_2.5_ and PM_10_, respectively. These greatly improved *QF* values are achieved through a series of inertial separation processes. The feature dimension of filtration channel is dozens of times larger than PM average size, which greatly decreases airflow resistance. Particularly, this inertial structure can be made of various types of materials, which shows great potential for low-cost fabrication of large-area devices. As a stand-alone device or incorporated with the existing PM air filter, this inertial impaction filter will bring great benefits to the public health.

## Introduction

**Figure 1:**
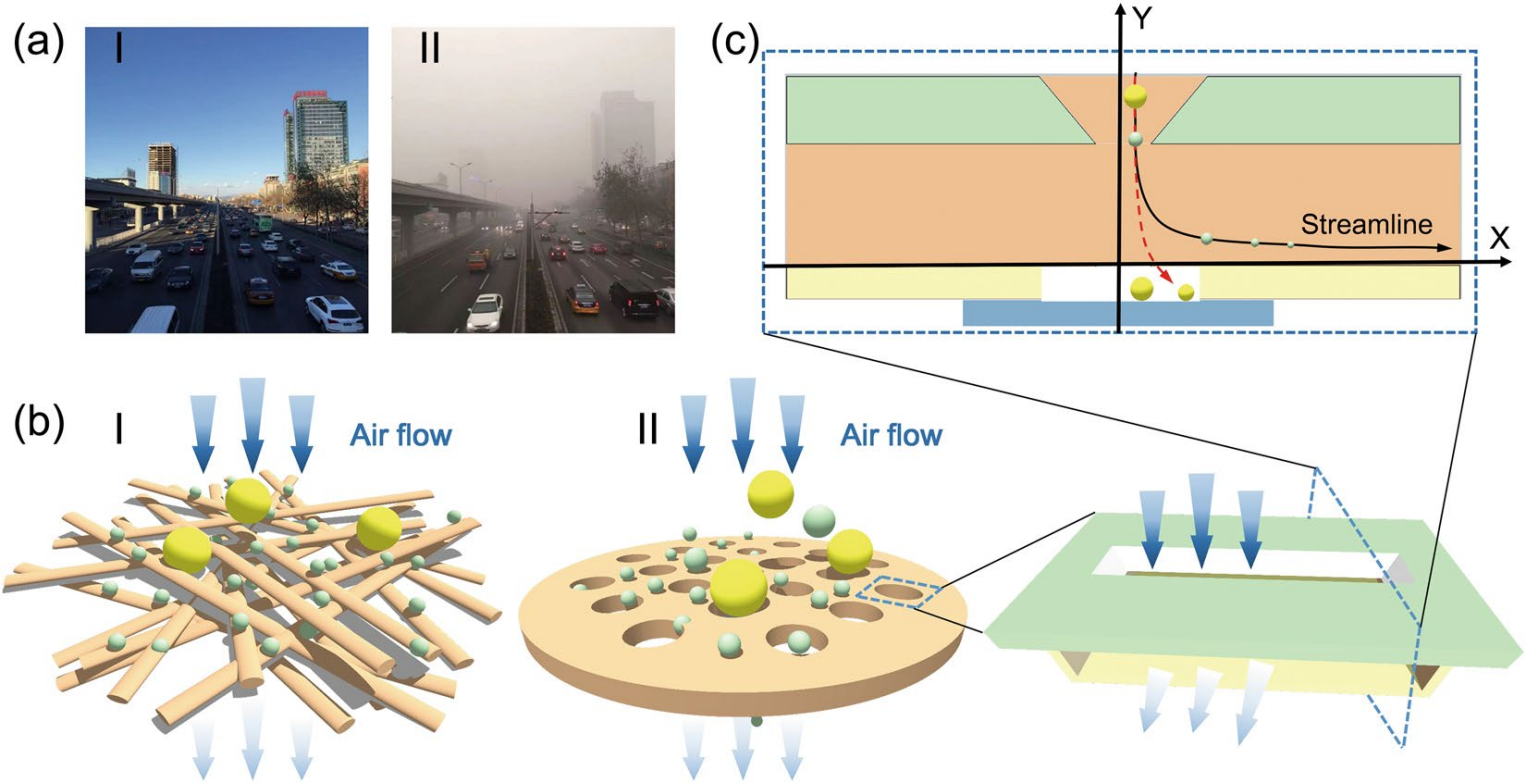
(**a**) Photographs of a random location in China during (I) a moderate day and (II) an extremely PM_2.5_-related hazardous day, respectively. (**b**) Conceptual sketches of two types of existing commercial air filters in common use: (I) a fibrous air filter with static electricity and (II) a membrane air filter with tiny pores. (**c**) Conceptual illustrations of the basic building block of an inertial impaction filter. Inset is the cross-sectional structure of an inertial impaction filter unit.

Air pollutions have raised serious concerns for the public health^1^. Among all the noxious pollutants suspended in air, airborne particulate matter (PM), especially particle with an aerodynamic diameter of less than or equal to 2.5 μm (referred as PM_2.5_), is the most hazardous air pollution since it can directly be inhaled into the parts of the lung through human nose and bronchi^2–4^. In particular, long-term exposure to PM air pollutions may result in various respiratory and cardiovascular diseases^5^. Over the past decade, PM air pollution problems become progressively worse, especially in major developing countries, such as China and India. These tiny particles primarily come from vehicle exhausts, coal combustion, biomass burning, and certain industrial processes, which are ever more abundant in most developing countries recently^5^. Until now, the control and removal of PM still remains a great challenge due to its small size and complex variation processes. Figure 1(a) shows two sharp contrast photographs of a location in China during a moderate day (I) and an extremely PM_2.5_-related hazardous day (II), respectively. During the hazardous air-quality days, the visibility is decreased dramatically and everyone will experience much more serious health effects. A recent report from the Global Burden of Disease (GBD) project of Institute for Health Metrics and Evaluation (IHME) and Health Effects Institute (HEI), indicates that ambient PM_2.5_ contributed to 4.2 million deaths globally and China (1.11 million) and India (1.1 million) each have the highest absolute numbers of deaths in 2015 due to extremely high levels of PM_2.5_ air pollution^6^.

So far, two main types of air filters have been in common use for removing PM particles in air^7^. One is a fibrous air filter, which captures PM particles by the combination of thick physical barriers and adhesion, as shown in Fig. 1(b) (I). In order to maximize filter efficiency, this type of air filter is usually very thick, which inevitably lead to the extremely low air flux with very high pressure drop. Besides, the rapidly weakening and disappearing of static electricity still severely hinders the advancement of this type of air filters with longer working life. Another type of air filter is a porous membrane filter based on size exclusion filtration, which is similar to a water filter, as shown in Fig. 1(b) (II). Generally, it can be fabricated by creating tiny pores on a solid substrate or thin film for filtering out PM particles with larger size. To obtain a high filter efficiency, the size of pores should be much smaller, which gives rise to high manufacturing costs and large airflow resistance. Nevertheless, for above two main types of existing commercial air filters, extraordinarily thick filtration channels or extremely tiny filtration pores can inevitably lead to the easy-to-block manners and extremely low air flux. Even worse, some hard PM particles containing a lot of carbon compounds or water molecules, will frequently impact and deform on filter surfaces during the filter process, seriously shortening the service life. Hence, the overall performance of existing air filter technologies, including PM removal efficiency, pressure drop, thermal stability, and service life, can rarely meet the requirements of high-performance air filters.

In order to improve the performance of current air filters, great efforts have been made recently. A series of nanofiber membranes, such as polyvinyl alcohol (PVA)^8^, polyacrylonitrile (PAN)^9–12^, polyethylene terephthalate (PET)^13^, polyurethane (PU)^14^, polylactic acid (PLA)^15^, polymide-66^16^, and polyimide (PI)^17^, are fabricated and developed to enable strong PM adhesion by effectively controlling the surface chemistry and microstructure of the air filters. Besides, various types of hybrid nanofibers, including PAN/silica^18^, polysulfide/titania^19^, and PLA/titania^20^, are constructed to achieve better performance via enhancing electrets effect or roughing surface of nanofibers. Despite recent progresses, these existing nanofiber filters still face the challenge of easy-to-block manner, poor thermal stability and high airflow resistance. Most recently reported low-cost air filters based on electro-spun nanofibers are still easily disabled or destroyed in the hot exhausts from automobile or factory^17^. For the existing filtration technologies, the forcing compromise between low airflow resistance and high filter efficiency still inevitably exists. To obtain a higher filter efficiency, the filtration pore need to be smaller than particle size while the filtration nanofiber need to be thicker than presently commercially available one. On the other hand, the tiny filtration pores or thick filtration channels has to cause higher airflow resistance, larger pressure drop, and the easy-to-block manner.

In order to resolve this contradiction fundamentally, here we demonstrate a conceptually new type of air filter for PM removal, which can work at high air flux with extremely low pressure drop. The conceptual illustration of this inertial filter is shown in Fig. 1(c). It can capture the PM particles suspended in air through a series of inertial separation processes. When a PM particle laden flow stream is caused to change direction suddenly, the PM particle with large mass will tend to follow an original trajectory due to the inertia effect. That is to say, traveling with hot exhausts, PM particles above a certain size possess so much inertial force that they cannot follow the air stream and finally impact with the adhesion layer which is used for capture. The concept of inertial impaction filter was firstly presented in 1955^21^. Over the years, various types of inertial impactors have been designed and developed, majority of which are low flow rate and designed for collection and monitoring of aerosol particles^22–32^. Compared with traditionally commercial air filters, the filter channel of inertial impaction filter can be greatly enlarged, even dozens of times larger than average PM particle size, which can modify the easy-to-block intrinsic manner of traditional air filters. Besides, this inertial impaction filter can be made of various types of materials, which shows great potential for low-cost fabrication of large-area devices and the excellent thermal endurance and stability.

The inset of Fig. 1(c) demonstrates the cross-sectional illustration of an inertial impaction filter structure. The PM particle moves over the filtration channel, not through it as in filtration. Larger-size filtration channels will effectively avoid blocking during filtration process and also cut down the resistance of air mass, which will greatly improve the airflow rate and effectively reduce the frequency of blocking. We anticipate that these low-cost and easy-to-use inertial impaction would greatly benefit the public health in preventing PM air pollution.

## Results and Discussions

According to the second law of Newton, the trajectory of a PM particle obeys the process that a particle mass multiplied by acceleration equals to the force exerted on it. In a fluid flow field, the PM particle will experience the Stokes drag force resulting from the relative velocity between particles and fluid flow field. Two-dimensional motion equations can be described by Equation 1 ^33^,

1 $$\begin{array}{c}\frac{Stk}{2}\frac{{d}^{2}x}{d{t}^{2}}=u-\frac{dx}{dt}\\ \frac{Stk}{2}\frac{{d}^{2}y}{d{t}^{2}}=v-\frac{dy}{dt},\end{array}$$

where (*x*, *y*) describes the instantaneous location of a particle at time *t* and (*u*, *v*) describes the instantaneous airflow velocity at point (*x*, *y*), respectively. The Stokes number ($Stk$) is a dimensionless number characterizing the behavior of particles suspended in a fluid flow field. In order to precisely describe the trajectories of incident particles, we calculate the instantaneous velocity (*u*, *v*) of related flow field at every point based on the Navier-Stokes equation at first. Near the stagnation point, the convective term in the Navier-Stokes equation will be neglected due to the low Reynolds number ($Re < 1$) and then the fluid’s Stokes equation may be solved by separation of variables. The instantaneous velocity of the flow field and related pressure at point (*x*, *y*) can be expressed as Equation 2. The detailed calculation is presented in Supplementary Material 1.

2 $$\begin{array}{c}u=8xy\\ v=-4{y}^{2}\\ P=-8uy,\end{array}$$

where (*u*, *v*) stands for the instantaneous velocity of the flow field and *P* stands for the pressure at point (*x*, *y*), respectively. After a simple substitution of Equation 1, the trajectory of an incident particle inside inertial impaction filter can be mathematically expressed as Equation 3,

3 $$\begin{array}{c}\frac{Stk}{2}\frac{{d}^{2}x}{d{t}^{2}}=8xy-\frac{dx}{dt}\\ \frac{Stk}{2}\frac{{d}^{2}y}{d{t}^{2}}=-4{y}^{2}-\frac{dy}{dt}.\end{array}$$

**Figure 2:**
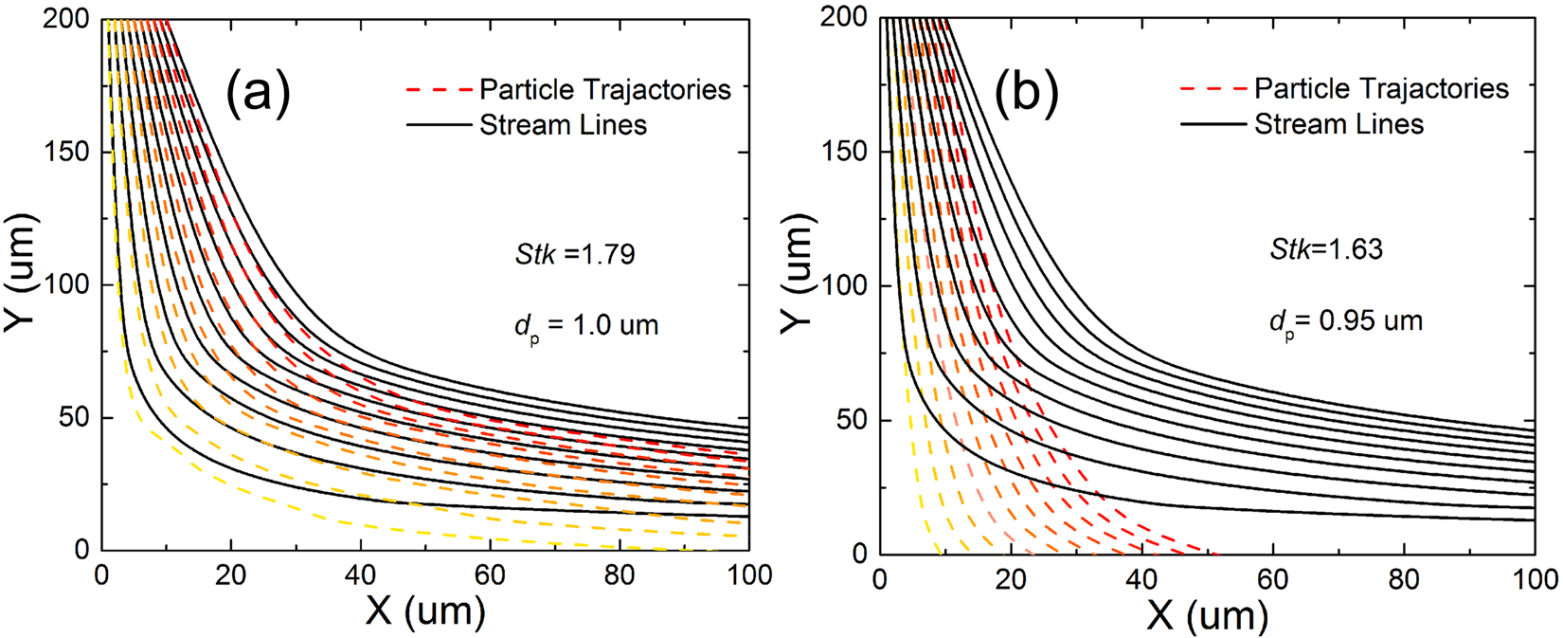
Particles’ trajectories (red dashed lines) and airflow streamlines (black solid lines) calculated by use of the Stokes flow field when the size of particles is 1.0 μm (**a**) and 0.95 μm (**b**), respectively.

According to the fluid-dynamic theory, a particle with a low *Stk* will follow fluid streamlines while a particle with a large *Stk* will be dominated by its inertia and continues along its initial trajectory^34^. *Stk* can be determined by Equation 4,

4 $$\begin{array}{c}Stk=\frac{Re}{9}(\frac{{\rho }_{P}}{{\rho }_{f}}+\frac{1}{2})\times {(\frac{{d}_{p}}{w})}^{2}\\ Re=\frac{inertial\,forces}{viscous\,forces}=\frac{{\rho }_{f}vw}{u}=\frac{uw}{\gamma },\end{array}$$

where ${\rho }_{P}$ stands for the particle density, ${\rho }_{f}$ stands for the density of air mass, ${d}_{p}$ stands for the average dimension of a particle, $u$ stands for the average inlet flow speed, $w$ stands for the width of chamber inlet and $\gamma$ stands for the kinematic viscosity of air mass, respectively. $Re$ is defined as the ratio of inertial forces to viscous forces and consequently quantifies the relative importance of these two types of forces for given airflow conditions. For the common PM particles, the particle density (${\rho }_{P}$) is estimated as 1000 kg/m^3^, the related air mass density (${\rho }_{f}$) is approximated as 1.29 kg/m^3^, and the kinematic viscosity of the fluid ($\gamma$) for air is 1.8 × 10^−5^ Pa/s, respectively^35^. As shown in Fig. 2, the trajectories of PM particles with different sizes are calculated according to Equation 3^33^. By use of an inertia impaction filter with the inlet width of 20 μm, under the airflow velocity of 7.7 m/s, all the particles with aerodynamic diameter larger than 1.0 μm ($Stk\ge 1.79$) can be removed by inertial effect while no particles with diameter smaller than 0.95 μm ($Stk\le 1.63$) impact with the collection plate. Through the precise control of the inlet width and related airflow velocity, the PM with sufficient inertia will slip across the sharply bending air streamlines and impact on adhesion layer while the finer particles will follow the bending air streamlines and move down stream of the impaction substrate. That is to say, traveling with hot exhausts, PM particles above a certain size possess so much mass and inertial force that they cannot follow the air stream and finally impact with the adhesion layer used for collection. By the similar calculation method, the relationship between incident velocity and designed width of chamber inlet can be concluded in Supplementary Material 2. Overall, the performance of an inertia filter can be predicted by numerical methods governing the fluid flow and particle motion. Several of analytical criterions about velocity have been developed for determining whether a particle is removed in Supplementary Material 3.

**Figure 3:**
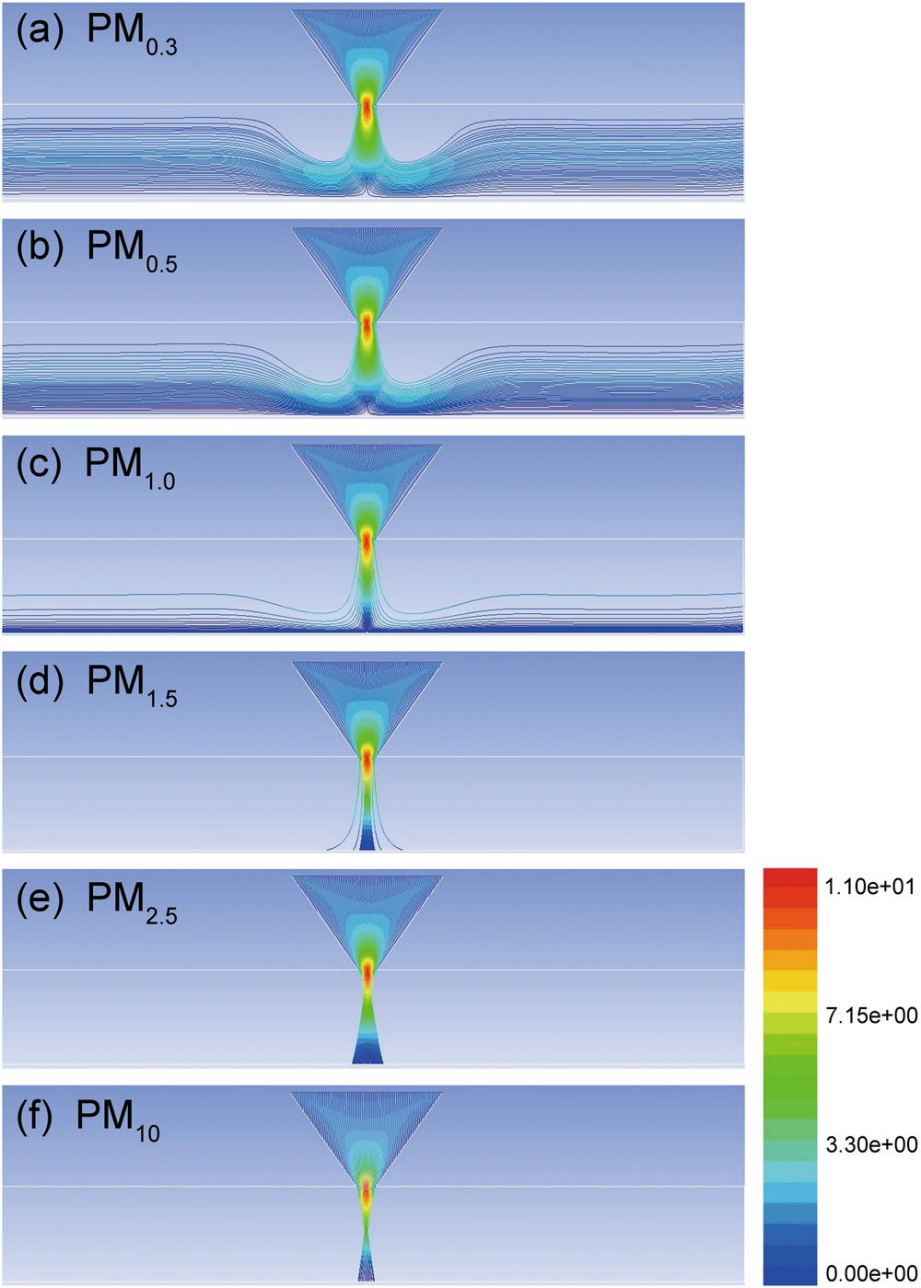
Trajectories of the PM particles with different sizes under the inlet velocity of 1.0 m/s.

In additions, Navier-Stokes equation and Continuity equation are built and solved by use of the commercial code FLUENT. For PM particles with different sizes, the trajectories inside an inertial impaction filter can be simulated and traced for 1.0 m/s inlet velocity as shown in Fig. 3 and for other inlet velocities as shown in Supplementary Material 4. Taking into account the gravity, Brownian motion, Saffman force, and pressure gradient, we calculate the related PM removal efficiency and pressure drop by use of the DPM model of FLUENT. With the increasing inlet velocities from 1.0 m/s to 8.0 m/s, both the PM removal efficiencies (Supplementary Material 5) and the pressure drop (Supplementary Material 6) are gradually increased, respectively. Simulation results show the pressure drop is less than 100 Pa even with the inlet velocity of up to 8.0 m/s, which indicates great potential for rapid PM removal with the relatively low pressure drop.

The real inertial impaction filters are fabricated by the standard microfabrication processes in Biomolecular Nanotechnology Center at University of California at Berkeley. Detailed fabrication procedures are depicted in Methods section. Note that the whole fabrication processes meet the industry standard and are cost-effective and mass production compatible. In our case, the micro-size nozzle is fabricated on Si wafers. For the convenience of fast prototyping, the out-flow channel and collection part are fabricated on the polydimethylsiloxane (PDMS) substrate by using the soft lithography technology. Particularly worth mentioning is the material of these inertia impaction filters is very replaceable-flexible. Hence, the whole fabrication process indicates great potential for the low-cost fabrication of large-area devices and the excellent thermal endurance and stability.

**Figure 4:**
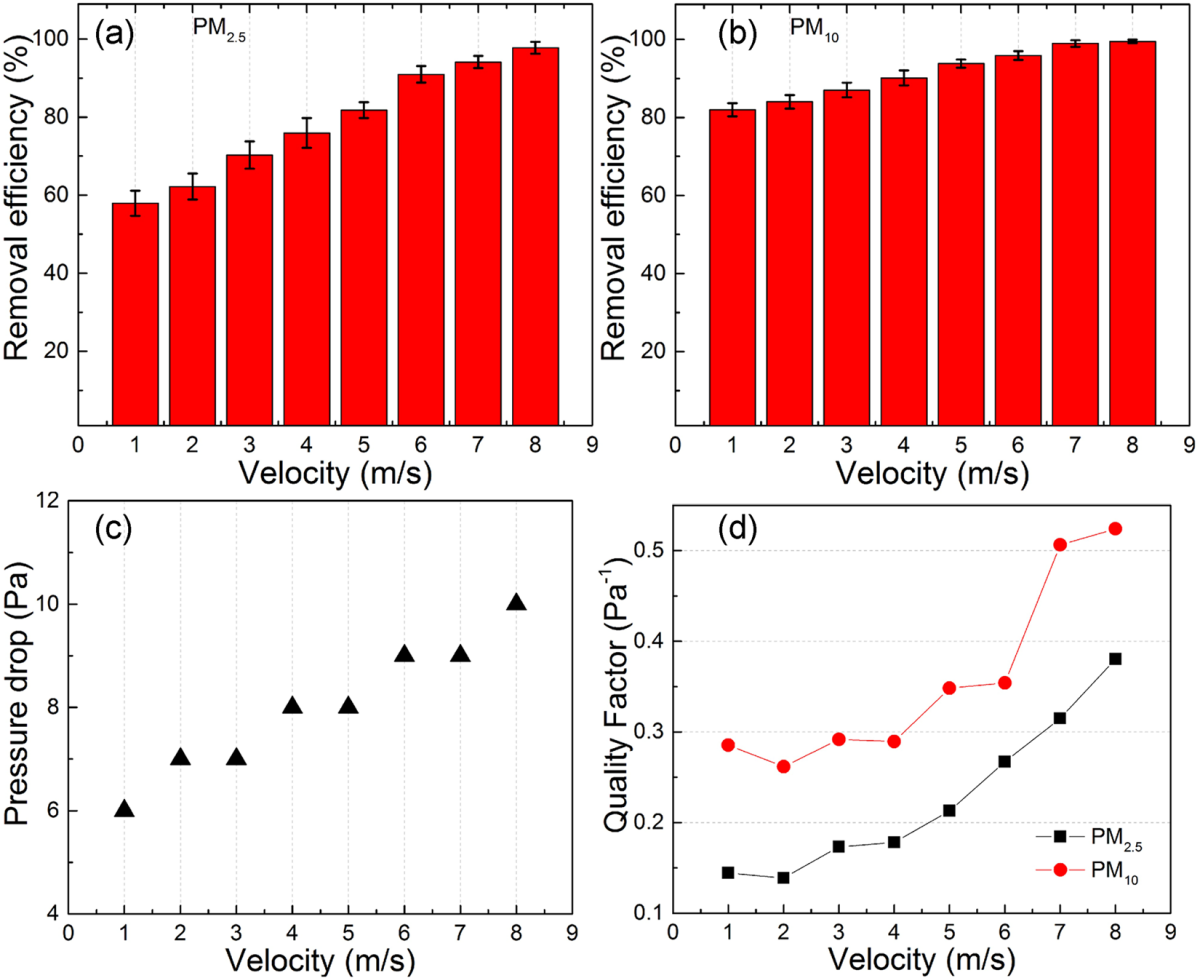
Removal efficiency of PM_2.5_ (**a**) and PM_10_ (**b**), pressure drop (**c**), and quality factor (**d**) of the inertial impaction filters under different airflow velocities. Error bar represents the standard deviation of three replicate measurements.

In order to evaluate the PM filtration performance, we design and fabricate a home-made measurement protocol. Detailed measuring process are depicted in Methods section. The PM source used here is generated by the burning of cigarettes and collected into a stainless steel reactor. The FT-IR analysis of the cigarette ashes confirms that the ingredients of smoke from burning cigarettes can be analogous to those of PM_2.5_ pollution (Supplementary Material 7). We consider that the cigarette ash illustrates a cognate spectrum to that of PM pollutants suspended in air. So in this work we choose the smoke from burning cigarettes as representative source PM. Note that the cigarette smoke PM particles have a wide size distribution from smallest size around 0.1 μm to largest size bigger than 10 μm, while the average size is 1.0 μm or less. With the increasing airflow velocity, both the removal efficiencies of PM_2.5_ and PM_10_ exhibit a slow increase as shown in Fig. 4(a) and (b) while the corresponding pressure drop curves shift upwards continuously as shown in Fig. 4(c). Under the airflow velocity of 8.0 m/s, Fig. 4(d) shows the inertial impaction filters show high PM removal efficiencies up to 97.77 ± 1.53% and 99.47 ± 0.45% for PM_2.5_ and PM_10_, respectively. Compared with the traditional air filters reported previously, the pressure drop is as low as 5–10 Pa. The extremely low pressure drop of our inertial impaction filter gives rise to greatly improved filtration performance, which can be evaluated regularly by a trade-off parameter named quality factor (*QF*)^36,37^. In comparison with the available literatures as shown in Table 1, our inertial impaction filter exhibits highly improved filtration performance achieved by largest *QF* values and extremely low pressure drop. We anticipate that there are no fundamental obstacles to extending these new techniques to PM filtrations at higher airflow velocity and this inertial impaction filter will be a start point for future low-cost, high-efficiency, and durable air filter with good thermos-stability, extremely low airflow resistance, and long operation life.

**Table 1:** A summary of performances of different materials and designs for PM filtration in recent literatures in comparison to what is achieved in this work.

Materials	*QF*^a^ value (Pa^−1^)	Δ*p*^b^ for different flow rates(Pa)	*E*^c^ (%)	Test particles	Additional advantages	Ref.
PAN nanofiber	0.024	133 Pa for 3 m/s	96.12	PM_2.5_	~85% transparency	^10^
PI nanofiber	0.1072	73 Pa for 0.2 m/s	99.97	PM_2.5_	~370 °C thermal stability	^17^
PAN nanofiber	0.1014	80 Pa for 0.2 m/s	99.97	PM_2.5_	~230 °C thermal stability	^17^
Nylon-6 nanofiber	0.062	42 Pa for 0.2 m/s	92.73	PM_2.5_	~10 times faster production speed	^38^
PAN nanofiber	0.052	124 Pa for 0.6~0.8 m/s	99.86	PM_2.5_	Large-scale direct coating	^39^
R-TENG^d^ enhanced PI nanofiber	—	17 Pa for 1 m/s 180 Pa for 5 m/s	97	PM	—	^40^
Nanofibrous MOF^e^	—	20 Pa for 50 mL/min	88.33 89.67	PM_2.5_ PM_10_	—	^41^
PVDF^f^ nanofibers with NIPS^g^	0.042	95 Pa for 0.0166 m/s	98.33	PM_2.5_	Releasing negative ions	^42^
PVDF/PAN	0.0954	45 Pa for 14 L/min	98.34	PM_2.5_	Rapidly transferring moisture	^43^
Commercial-1	0.00062	299 Pa	16.93	PM_2.5_	—	^10^
Commercial-2	0.0068	809 Pa	99.58	PM_2.5_	—	^10^
This work	0.380 0.524	10 Pa for 8.0 m/s	97.77 99.47	PM_2.5_ PM_10_	Extremely low airflow resistance and pressure drop	

^a^*QF* stands for the quality factor, *QF* = −ln(1 − *E*%)/Δ*p*.

^b^Δ*p* stands for the pressure drop.

^c^*E* stands for the PM removal efficiency.

^d^R-TENG stands for the rotating triboelectric nano generator.

^e^MOF stands for the metal organic framework.

^f^PVDF stands for the polyvinylidene fluoride.

^g^NIPs stands for the negative ions powders.

In summary, the design and fabrication process of a conceptually new type of air filter is demonstrated in detail. Under the airflow velocity of 8.0 m/s, the real inertial impaction filters show high PM removal efficiencies of up to 97.77 ± 1.53% and 99.47 ± 0.45% for PM_2.5_ and PM_10_, respectively. Compared with traditional filters, inertia impaction filters also exhibit extremely low pressure drop of 5–10 Pa and greatly improved quality factor values of up to 0.380 Pa^−1^ for PM_2.5_ and 0.524 Pa^−1^ for PM_10_, respectively. Meanwhile, the analysis and simulation of the filter efficiency and pressure drop paves the way for future research into designs of inertia impaction filters. In particular, this inertial structure can be made of various types of materials with excellent thermal endurance and stability, which shows great potential for low-cost fabrication of large-area devices. We consider that these inertia impaction filters can work both independently and work together with traditional filters as a pretreatment equipment to achieve a healthier public living environment.

## Methods

The real inertial impaction filters are fabricated by the standard microfabrication techniques, as depicted in Supplementary Material 8. For the nozzle part of inertial impaction filter, fabrication process begins with prime grade, double-sided polished, ultrathin Si wafers with < 100 > orientation. Starting from cleaning by Piranha, wafers are air dried and deposited stoichiometric silicon nitride (SiN_x_) by LPCVD method on both sides. Next, photolithography process is carried out to pattern the shape of nozzle. When the pattern is generated, CF_4_/O_2_ plasma etching is conducted to generate SiN_x_ mask by use of patterned photoresist as a protect layer. Then, the photoresist is stripped away by soaking in 90 °C heated PRS-3000 bath. Wafers are cleaned and dried by N_2_ gun after stripping. Subsequently, the nozzle is generated by use of KOH solution etching. KOH bath has a great selectivity toward SiN_x_ and Si so the SiN_x_ mask kept well during wet etching. After wet etching, wafers will soak into HCl/H_2_O_2_ solution to remove metal ions for 2 hours and then be washed by deionized water and dried by N_2_ gun again. Next, the residual SiN_x_ is removed by CF_4_/O_2_ plasma etching. Served to exhaust the gas stream, the bottom part is made by use of soft lithography technology. Finally, the Si-based nozzle and PDMS-based bottom part are assembled together.

As depicted in Supplementary Material 9, the inertial impaction filter is placed in the air inlet and sealed with double-sided adhesive tape to make sure the source PM passing into the transparent chamber completely. PM particle number concentrations are detected by use of a professional PM counter (PureAir, China) placed inside of transparent chamber, and the pressure drop is measured by use of an electronic pressure transmitters. The removal efficiency is defined by comparing the particle number concentrations before and after filtration, and the airflow velocity is tested by an anemometer.

## Electronic supplementary material

Supplementary Materials
