# Supplementary material for: High-performance inertial impaction filters for particulate matter removal
Source: Sci Rep. 2018 Mar 19;8:4757. doi: 10.1038/s41598-018-23257-x (PMC5859014; doi:10.1038/s41598-018-23257-x)
Supplement: Supplementary file 1 — Supplementary Materials [file 41598_2018_23257_MOESM1_ESM.pdf]

Supplementary Materials

High-performance inertial impaction filters for particulate matter removal

Xiaowei Zhang[1,2,+], Wei Zhang[3,+], Mingqiang Yi[4], Yingjie Wang[1], Pengjun Wang[1,*], Jun Xu[2,*], Fenglei Niu[3,*], and Feng Lin[5]

[1] Department of Electrical Engineering and Computer Science, Ningbo University, Ningbo, 315211, China

[2] National Laboratory of Solid State Microstructures, Collaborative Innovation Centre of Advanced Microstructures, and Department of Electronic Science and Engineering, Nanjing University, Nanjing, 210093, China

[3] Beijing Key Laboratory of Passive Nuclear Power Safety and Technology, North China Electric Power University, Beijing, 102206, China

[4] Microfluidic Foundry LLC, San Pablo, CA, 94806, United States

[5] Department of Chemistry, Virginia Tech, VA, 24061, United States

+ these authors contributed equally to this work

* wangpengjun@nbu.edu.cn , junxu@nju.edu.cn , niufenglei@ncepu.edu.cn

Table of Contents

1. Detailed calculation of the instantaneous velocity of the flow field and pressure

2. Relationship between exhaust velocity and designed width of chamber inlet.

3. Requirements of PM filtrations on velocity for removal

4. Particle’s trajectories with the different inlet velocities based on FLUENT simulation

5. PM removal efficiency with the increasing inlet velocities from 1 m/s to 8 m/s

6. Pressure drops with the increasing inlet velocities from 1 m/s to 8 m/s

7. Fourier transform infrared spectroscopy (FT-IR) spectrum of the cigarette ashes

8. Scheme for the fabrication procedures of the as-made inertial impaction filter

9. Photograph showing the setup of PM filtration efficiency measurement

1. Detailed calculation of the instantaneous velocity of the flow field and pressure

The chamber inlet of an inertial impaction filter has a rectangular cross section of width (w) and length (l). For easier and more accurate theoretical predictions on the collection performance, we design with $l \gg w$ to approximate the inlet as a two-dimensional nozzle. For the stream function $\varphi(r,\theta)$, $\nabla^4\varphi = 0$ admits a separation of variable solution in plane polar coordinates (r,θ) centered at "0" of the form.[1,2]

$$\varphi = r^{\lambda} \times \left[A_{\lambda}\cos\lambda\theta + B_{\lambda}\sin\lambda\theta + C_{\lambda}\cos(\lambda-2)\theta + D_{\lambda}\sin(\lambda-2)\theta\right], \qquad \text{(S1)}$$

where A_{λ}, B_{λ}, C_{λ}, D_{λ} are constants and λ is the eigenvalue, respectively. The following boundary conditions,

$$u_{\lambda} = u_{\theta} = 0 \ \ when\ \theta = 0;$$

$$u_{\theta} = 0\ and\ \frac{\partial u_r}{\partial\theta} = 0\ when\ \theta = \frac{\pi}{2}, \qquad \text{(S2)}$$

can be applied. For the leading-order term, the velocity components can be expressed by the following Equation S3 and S4, respectively.

$$u_r = Cr^2(3\times\sin3\theta - \sin\theta) + Br^3(\cos4\theta - \cos2\theta) + O(r^4), \qquad \text{(S3)}$$

$$u_{\theta} = 3\times Cr^2(\cos3\theta - \cos\theta) + Br^3(-\sin4\theta + 2\times\sin2\theta) + O(r^4), \qquad \text{(S4)}$$

where $0 \le \theta \le \frac{\pi}{2}$ and $r \to 0^{+}$ (cylindrical coordinates). In the above equations, $\lambda=1$ and $\lambda=2$ are inadmissible because they will result in a pressure singularity at $r=0$ due to $\nabla P = u\nabla^2 u$ ($P \sim \frac{1}{r}\ when\ \lambda=1$; $P \sim \ln r\ when\ \lambda=1$) while $\lambda=3$ and $\lambda=4$ are adopted. Changing back to the Cartesian coordinates,

$$u = 8xy$$
$$v = -4y^2 \ , \qquad \text{(S5)}$$
$$P = -8uy$$

where (u, v) stands for the instantaneous velocity of the flow field and P stands for the pressure at point (x, y), respectively. The above analyses indicate that in the vicinity of the collection hole, the velocity is normal to the inlet of the collection hole and the pressure varies with the square of the normal distance to the inlet of the collection hole and horizontal distance from the center of inertia filter, respectively.

2. Relationship between exhaust velocity and designed width of chamber inlet

Table S1. Relationship between various exhaust velocity and designed width of chamber inlet for collection of 1.0 μm PM particles

Incident exhaust velocity (m/s)	Width of chamber inlet (um)
1.0	10.8
2.0	12.2
3.0	13.4
4.0	14.4
5.0	16.6
6.0	18.6
7.0	20.2
7.7	20.4

3. Requirements of PM filtrations on velocity for removal

In many cases, it is only necessary to decide whether a particle could actually be separated, given its position and velocity components at a point

sufficiently close to the plate. In such cases, an analytical criterion on velocity can be developed for determining whether a particle is removed. Our asymptotic solution for the flow field near the stagnation point suggests that the velocity decreases as a quadratic function of y. A linearly decreasing flow field velocity would require a nonphysical pressure singularity at the wall.[3] Therefore, it's necessary to develop different criteria to judge whether a particle impacts or not. We assume the vertical velocity of particle is v_0 at $y = y_0$, and the fluid's velocity is v_{1f} at $y = \Delta$.

1) Simplest (most conservative collection criterion).

Considering Equation 1 and neglecting the quadratic term, we obtain that, for the particle to arrive at the wall, it needs,

$$-v_0 > \frac{2y_0}{Stk}. \tag{S6}$$

2) Results based on Stokes flow near the wall.

We assume fluid velocity of the form $v = v_{1f}\left(\frac{y}{\Delta}\right)^2$, the collection criterion is,

$$-v_0 > \frac{2y_0}{Stk} - \frac{4}{3Stk}\frac{\mathrm{v}_{1f}y_0{}^3}{\mathrm{v}_0\Delta^2} = \frac{2y_0}{Stk}(1 - \frac{2}{3}\frac{\mathrm{v}_{1f}y_0{}^2}{\mathrm{v}_0\Delta^2}). \tag{S7}$$

3) When pressure effects are considered, the collection criterion is,

$$\frac{Stk}{2}\frac{d^2y}{dt^2} = v_{1f}\left(\frac{y}{\Delta}\right)^2 - \frac{dy}{dt} - \frac{1}{9}\left(\frac{d_p}{W}\right)^2\frac{\mathrm{v}_{1f}}{\Delta^2}. \tag{S8}$$

Notice that additional work needs to be done to overcome the pressure gradient as the flow approaches the stagnation point. Therefore, a higher initial speed is needed for the particle to reach the wall. The collection criterion is,

$$-v_0 > \frac{2y_0}{Stk}[1 - \frac{2v_{1f}y_0^2}{3v_0\Delta^2} + \frac{2v_{1f}}{9v_0\Delta^2}\left(\frac{d_p}{W}\right)^2]\ . \tag{S9}$$

4. Particle's trajectories with the different inlet velocities based on FLUENT simulation.

1) Trajectories of PM particles with different sizes under the inlet velocity of 2 m/s.

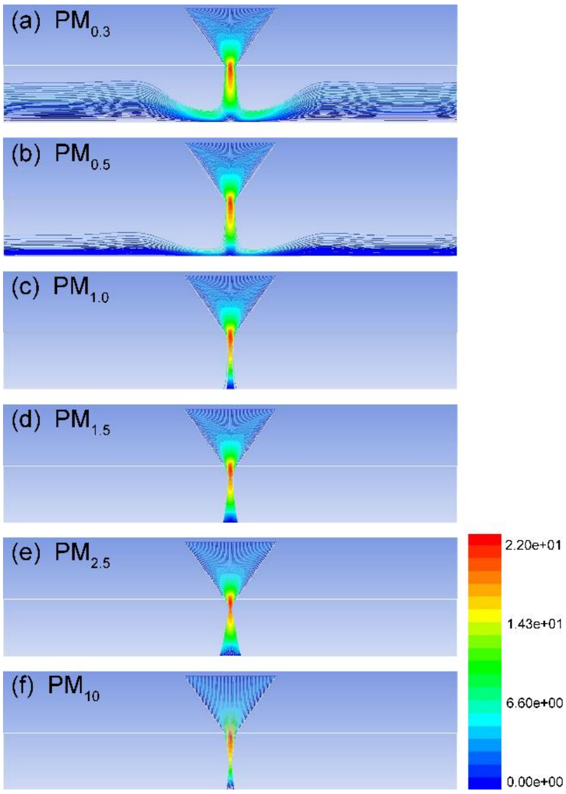

Figure S1. Trajectories of PM particles under the inlet velocity of 2 m/s.

2) Trajectories of PM particles with different sizes under the inlet velocity of 3 m/s.

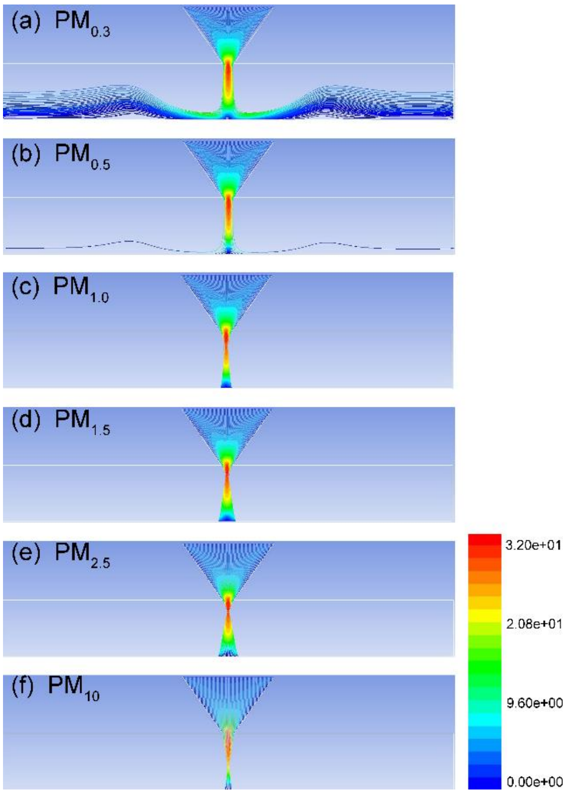

Figure S2. Trajectories of PM particles under the inlet velocity of 3 m/s.

3) Trajectories of PM particles with different sizes under the inlet velocity of 4 m/s.

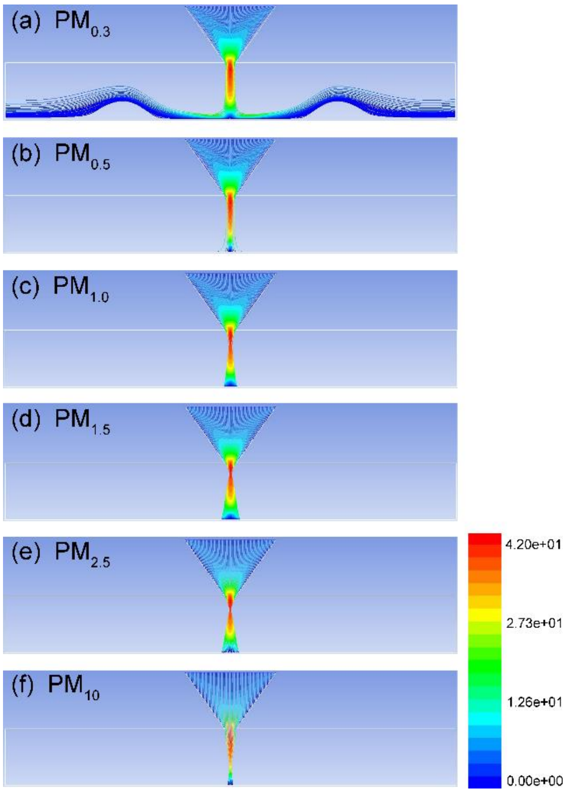

Figure S3. Trajectories of PM particles under the inlet velocity of 4 m/s.

4) Trajectories of PM particles with different sizes under the inlet velocity of 5 m/s.

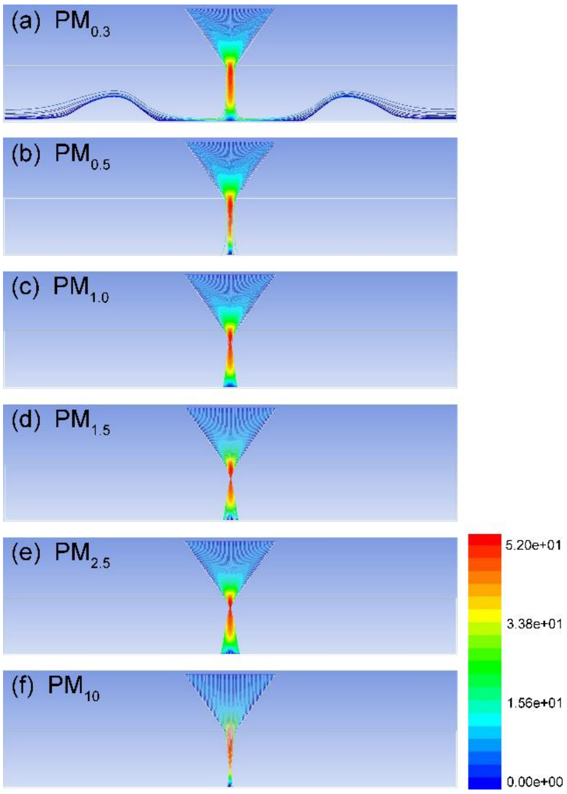

Figure S4. Trajectories of PM particles under the inlet velocity of 5 m/s.

5) Trajectories of PM particles with different sizes under the inlet velocity of 6 m/s.

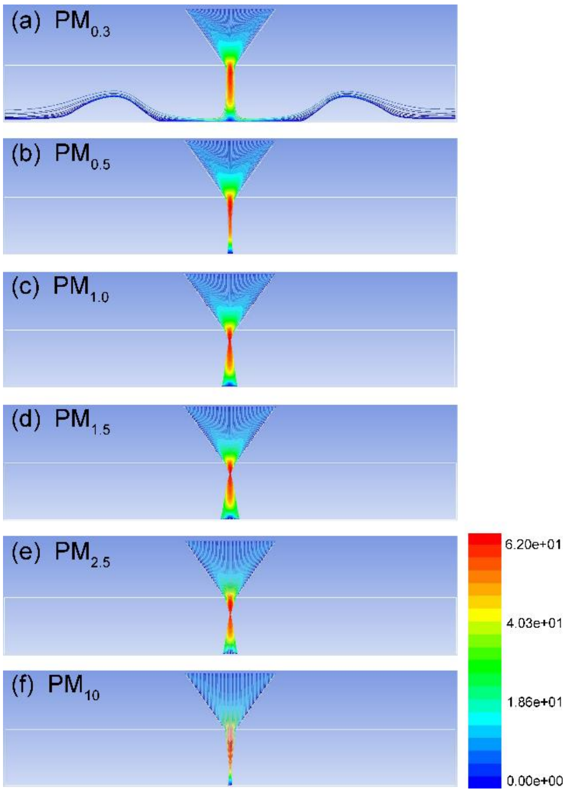

Figure S5. Trajectories of PM particles under the inlet velocity of 6 m/s.

6) Trajectories of PM particles with different sizes under the inlet velocity of 7 m/s.

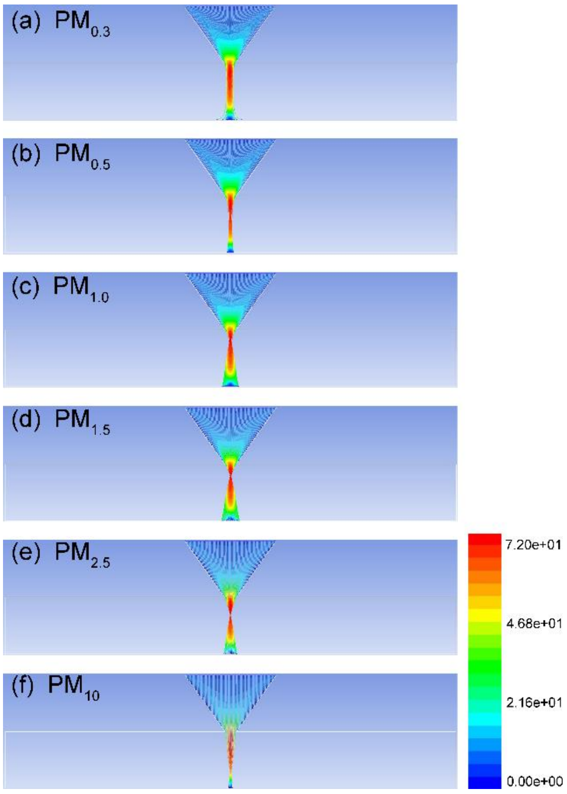

Figure S6. Trajectories of PM particles under the inlet velocity of 7 m/s.

7) Trajectories of PM particles with different sizes under the inlet velocity of 8 m/s.

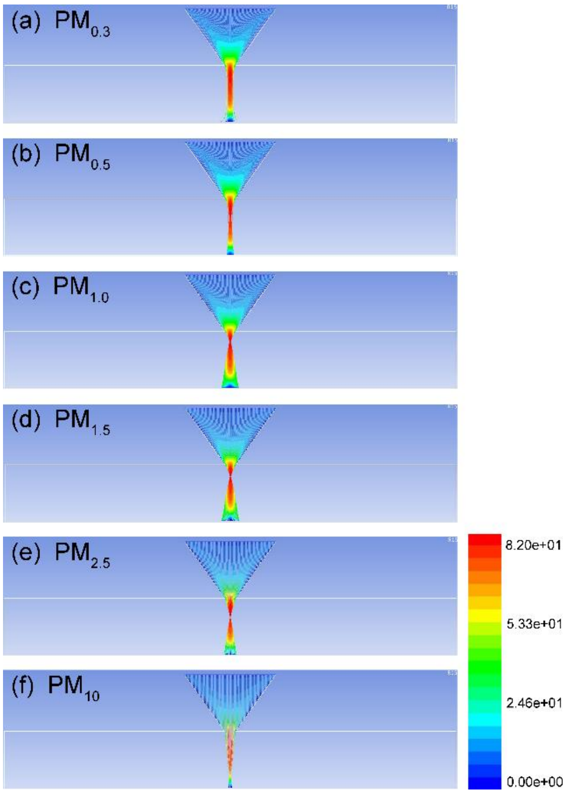

Figure S7. Trajectories of PM particles under the inlet velocity of 8 m/s.

5. PM removal efficiency with the increasing inlet velocities from 1m/s to 8m/s

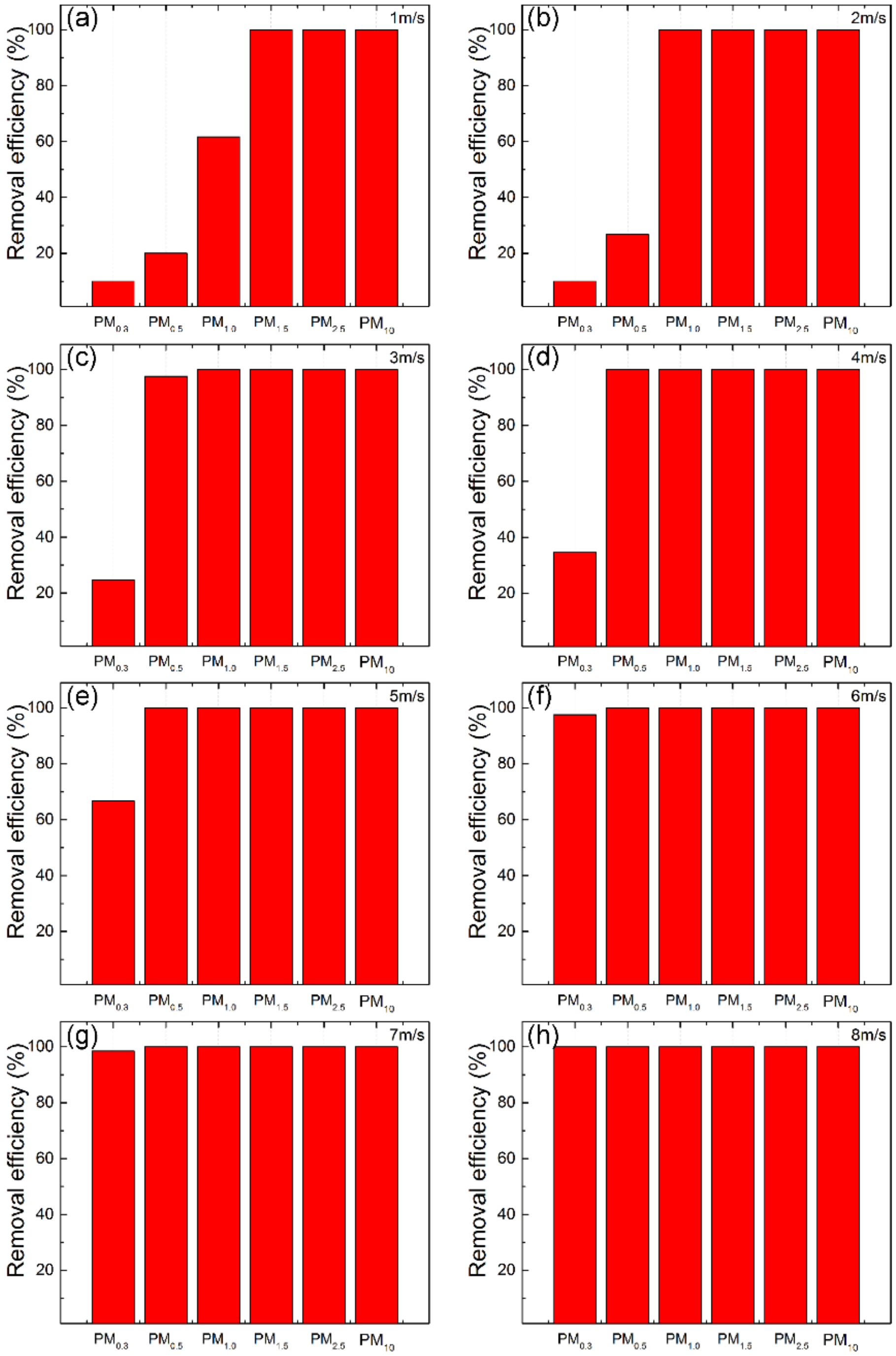

Figure S8. Simulations on removal efficiency of PM particles with different sizes for inlet velocities from 1m/s to 8m/s: (a) 1 m/s; (b) 2 m/s; (c) 3 m/s; (d) 4 m/s; (e) 5 m/s; (f) 6m/s; (g) 7m/s; (h) 8m/s.

6. Pressure drops with the increasing inlet velocities from 1m/s to 8m/s

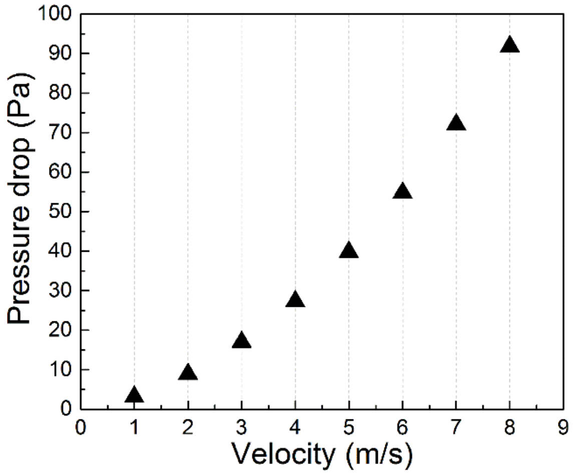

Figure S9. Simulations on pressure drops with the different inlet velocities.

7. Fourier transform infrared spectroscopy (FT-IR) spectrum of the cigarette ashes.

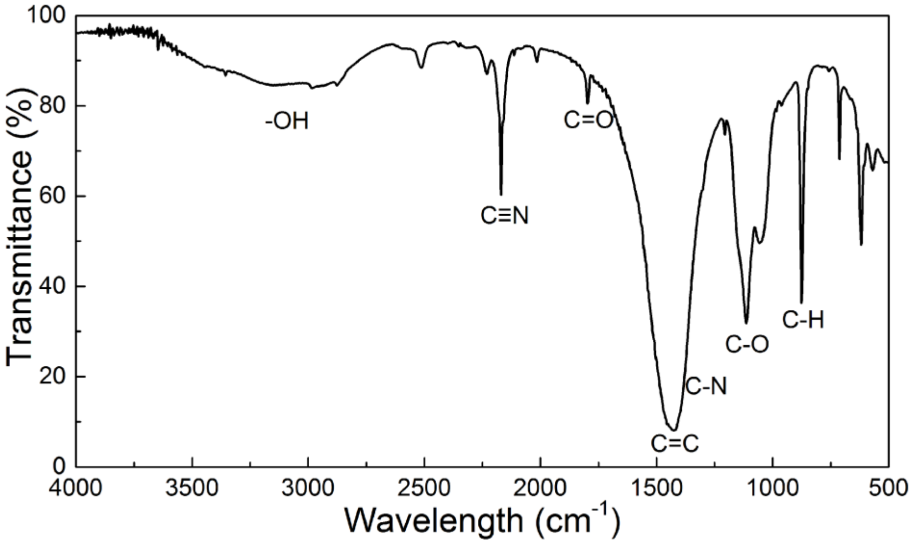

Figure S10. Fourier transform infrared spectroscopy (FT-IR) spectrum of the cigarette ashes.

As shown in Figure S10, the FT-IR spectrum of cigarette ashes illustrates a series of remarkable peaks at 2173 cm^{-1}, 1793 cm^{-1}, and 1421 cm^{-1}, which demonstrates the existence of C≡N, C=O, C=C, and C—N bonds. The peaks at 1115 cm^{-1} and 1053 cm^{-1} are ascribed to the stretching vibrations of C—O bonds while the peak at 871 cm^{-1} is ascribed to the out-of-plane bending vibrations of C—H linked to the benzene ring.[4,5] Functional groups, such as C=O, C—O, and C—N bonds, are also distributed on the surfaces of PM

particles. The FT-IR results confirm that the ingredients of smoke from burning cigarettes can be analogous to those of $PM_{2.5}$ pollution according to the previous report.[6]

We consider that the cigarette ash illustrates a cognate spectrum to that of $PM_{2.5}$. So in this work we choose the smoke from burning cigarettes as representative source PM. Note that the cigarette smoke PM particles have a wide size distribution from smallest size around 0.1 μm to largest size bigger than 10 μm, while the average size is 1.0 μm or less.[6]

8. Scheme for the fabrication procedures of the as-made inertial impaction filter

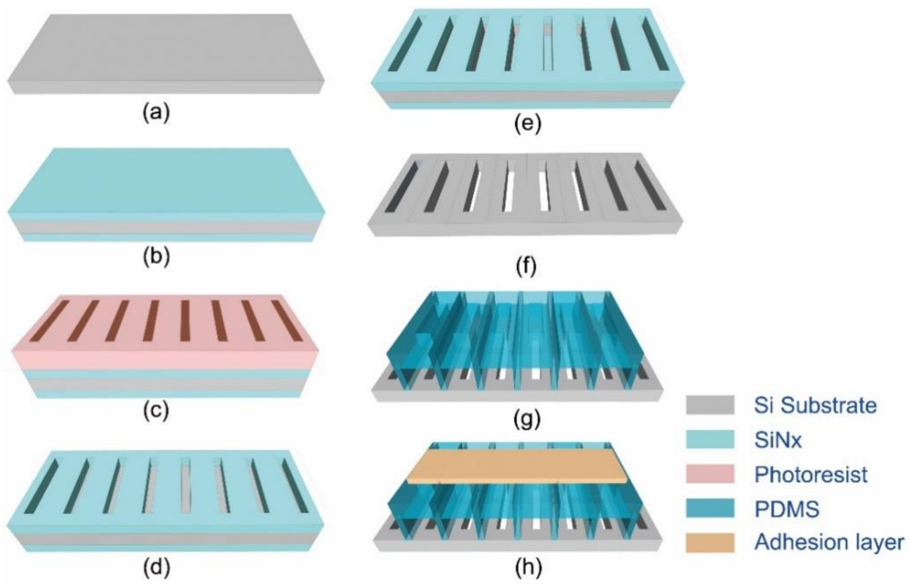

Figure S11. Scheme for the fabrication procedures of the as-made inertial impaction filter.

9. Photograph showing the setup of PM filtration efficiency measurement

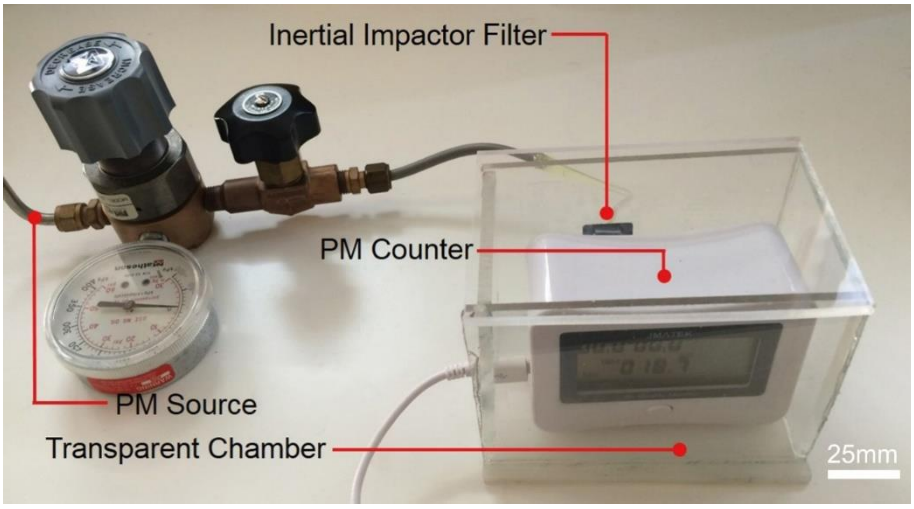

Figure S12. Photograph showing the setup of PM filtration efficiency measurement. The removal efficiency is defined by comparing the particle number concentrations before and after filtration.

References

1. Marple, V. A., Liu, B. Y. H. & Whitby, K. T. On the flow fields of inertial impactors. *J. Fluids Eng.* **96(4)**, 394-400 (1974).

2. Perlekar, P., Pal, N. & Pandit, R. Two-dimensional Turbulence in Symmetric Binary-Fluid Mixtures: Coarsening Arrest by the Inverse Cascade. *Sci. Rep.* **7**, 44589 (2017).

3. Niu, F., Du, X., Qi, H., Yi, M. & Yang, X. Modeling analyses of radioactive aerosol flow and collection in mesoscopic impactor filters. *Prog. Nucl. Energ.* **88**, 147-155 (2016).

4. Liu, C., Hsu, P., Lee, H., Ye, M., Zheng, G., Liu, N., Li, W. & Cui, Y. Transparent air filter for high-efficiency $PM_{2.5}$ capture. *Nat. Commun.* **6**, 6205 (2015).

5. Gao, H. C., Yang, Y. Q., Akampumuza, O., Hou, J., Zhang, H. N. & Qin, X. H. A low filtration resistance three-dimensional composite membrane fabricated via free surface electrospinning for effective $PM_{2.5}$ capture. *Environ. Sci.: Nano.* **4**, 864-875 (2017).

6. Pipal, A. S., Kulshrestha, A. & Taneja, A. Characterization and morphological analysis of airborne $PM_{2.5}$ and PM_{10} in Agra located in north central India. *Sci. Total Environ.* **45**, 3621-3630 (2011).
